# Supplementary material for: Content-qualified antenatal care coverage in Lesotho: An ordinal logistic regression analysis of the 2023–2024 demographic and health survey
Source: PLoS One. 2026 May 15;21(5):e0348679. doi: 10.1371/journal.pone.0348679 (PMC13178882; doi:10.1371/journal.pone.0348679)
Supplement: Attachment — Submitted filename: PONE-D-26-05429-AF.pdf [file pone.0348679.s002.pdf]

PLOS One

Content-qualified antenatal care coverage in Lesotho: an ordinal logistic regression analysis of the 2023-2024 demographic and health survey

--Manuscript Draft--

**Manuscript Number:**	PONE-D-26-05429
**Article Type:**	Research Article
**Full Title:**	Content-qualified antenatal care coverage in Lesotho: an ordinal logistic regression analysis of the 2023-2024 demographic and health survey
**Short Title:**	Content-qualified antenatal care coverage in Lesotho
**Corresponding Author:**	Kindu Yinges Wondie, MSc<br>University of Gondar<br>Gondar, Amhara Regional state ETHIOPIA
**Keywords:**	Content-qualified, Content-qualified Antenatal Care, Antenatal Care, Prenatal Care, Lesotho, Sub-Saharan Africa
**Abstract:**	Background: Despite high antenatal care contact coverage in Lesotho, the content and quality of care remains suboptimal. This study assessed the level and determinants of content-qualified antenatal care coverage using the 2023-2024 Lesotho Demographic and Health Survey.<br>Methods: Data were analyzed from 1,112 women aged 15-49 who had a live birth or stillbirth in the 2 years preceding the survey. Content-qualified antenatal care was constructed as a composite score (0-16) incorporating the timing of initiation, number of contacts, attendance of skilled providers, and receipt of 10 key content components. The content-qualified antenatal care coverage score was categorized into ordered tertiles (low: 0-12, moderate: 13, high: 14-16). Ordinal logistic regression (proportional odds model) was used to identify factors associated with higher content-qualified antenatal care categories, accounting for complex survey design.<br>Results: Overall, 71.9% of women had moderate or high ANCq scores. Higher content qualified antenatal care coverage was significantly associated with being married or living with a partner, having secondary or higher education (AOR = 2.05 and 1.90), using the internet at least once per week (AOR = 1.61), having a wanted pregnancy (AOR = 2.87), living in a smaller household (AOR = 1.36), and residing in a community with a higher level of education (AOR = 1.38).<br>Conclusion: Despite reasonable antenatal care contact coverage, content-qualified antenatal care coverage remains low and uneven. To close the coverage gap, targeted interventions to improve maternal education, internet access, pregnancy planning, and community education are essential.
**Order of Authors:**	Kindu Yinges Wondie, MSc
	Alemneh Tadesse Kassie, MSc
	Nuhamin Tesfa Tsega, MSc
	Berihun Agegn Mengistie, MSc
	Endalk Birrie Wondifraw, MSc
	Getie Mihret Aragaw, MSc
**Opposed Reviewers:**	
**Additional Information:**	
**Question**	**Response**
**Financial Disclosure**<br><br>Enter a financial disclosure statement that describes the sources of funding for the work included in this submission. Review	The author(s) received no specific funding for this work.

Powered by Editorial Manager® and ProduXion Manager® from Aries Systems Corporation

the submission guidelines for detailed requirements. View published research articles from *PLOS ONE* for specific examples.<br><br>This statement is required for submission and **will appear in the published article** if the submission is accepted. Please make sure it is accurate.<br><br>**Funded studies**<br>Enter a statement with the following details:<br>• Initials of the authors who received each award<br>• Grant numbers awarded to each author<br>• The full name of each funder<br>• URL of each funder website<br>• Did the sponsors or funders play any role in the study design, data collection and analysis, decision to publish, or preparation of the manuscript?<br><br>Did you receive funding for this work?	
**Competing Interests**<br><br>Use the instructions below to enter a competing interest statement for this submission. On behalf of all authors, disclose any competing interests that could be perceived to bias this work—acknowledging all financial support and any other relevant financial or non-financial competing interests.<br><br>This statement is required for submission and **will appear in the published article** if the submission is accepted. Please make sure it is accurate and that any funding sources listed in your Funding Information later in the submission form are also declared in your Financial Disclosure statement.<br><br>View published research articles from *PLOS ONE* for specific examples.	The authors have declared that no competing interests exist.

**NO authors have competing interests**<br><br>Enter: *The authors have declared that no competing interests exist.*<br><br>**Authors with competing interests**<br><br>Enter competing interest details beginning with this statement:<br><br>*I have read the journal's policy and the authors of this manuscript have the following competing interests: [insert competing interests here]*<br><br>* typeset	
**Ethics Statement**<br><br>Enter an ethics statement for this submission. This statement is required if the study involved:<br><br>• Human participants<br>• Human specimens or tissue<br>• Vertebrate animals or cephalopods<br>• Vertebrate embryos or tissues<br>• Field research<br><br>Write "N/A" if the submission does not require an ethics statement.<br><br>General guidance is provided below. Consult the submission guidelines for detailed instructions. **Make sure that all information entered here is included in the Methods section of the manuscript.**	This study is a secondary analysis of publicly available, anonymized Demographic and Health Surveys data. The current analysis was formally approved by the DHS Program, which granted written and signed ethical approval for the use of the data. Since the dataset contains no identifying information, it was deemed exempt from further ethical review. The protocol for the 2023–24 LDHS received clearance from both the ICF Institutional Review Board ethics committee and the Lesotho Ministry of Health Research and Ethics Committee.

Powered by Editorial Manager® and ProduXion Manager® from Aries Systems Corporation

**Format for specific study types**<br><br>**Human Subject Research (involving human participants and/or tissue)**<br>• Give the name of the institutional review board or ethics committee that approved the study<br>• Include the approval number and/or a statement indicating approval of this research<br>• Indicate the form of consent obtained (written/oral) or the reason that consent was not obtained (e.g. the data were analyzed anonymously)<br><br>**Animal Research (involving vertebrate animals, embryos or tissues)**<br>• Provide the name of the Institutional Animal Care and Use Committee (IACUC) or other relevant ethics board that reviewed the study protocol, and indicate whether they approved this research or granted a formal waiver of ethical approval<br>• Include an approval number if one was obtained<br>• If the study involved *non-human primates*, add *additional details* about animal welfare and steps taken to ameliorate suffering<br>• If anesthesia, euthanasia, or any kind of animal sacrifice is part of the study, include briefly which substances and/or methods were applied<br><br>**Field Research**<br><br>Include the following details if this study involves the collection of plant, animal, or other materials from a natural setting:<br>• Field permit number<br>• Name of the institution or relevant body that granted permission	
**Data Availability**<br><br>Authors are required to make all data underlying the findings described fully available, without restriction, and from the time of publication. PLOS allows rare exceptions to address legal and ethical concerns. See the PLOS Data Policy and FAQ for detailed information.	Yes - all data are fully available without restriction

A Data Availability Statement describing where the data can be found is required at submission. Your answers to this question constitute the Data Availability Statement and **will be published in the article**, if accepted.<br><br>**Important:** Stating 'data available on request from the author' is not sufficient. If your data are only available upon request, select 'No' for the first question and explain your exceptional situation in the text box.<br><br>Do the authors confirm that all data underlying the findings described in their manuscript are fully available without restriction?	
**Describe where the data may be found in full sentences. If you are copying our sample text, replace any instances of XXX with the appropriate details.**<br><br>• If the data are **held or will be held in a public repository**, include URLs, accession numbers or DOIs. If this information will only be available after acceptance, indicate this by ticking the box below. For example: *All XXX files are available from the XXX database (accession number(s) XXX, XXX.).*<br>• If the data are all contained **within the manuscript and/or Supporting Information files**, enter the following: *All relevant data are within the manuscript and its Supporting Information files.*<br>• If neither of these applies but you are able to provide **details of access elsewhere**, with or without limitations, please do so. For example:<br><br>*Data cannot be shared publicly because of [XXX]. Data are available from the XXX Institutional Data Access / Ethics Committee (contact via XXX) for researchers who meet the criteria for access to confidential data.*<br><br>*The data underlying the results presented in the study are available from (include the name of the third party*	he data underlying the results presented in the study are available from https://www.dhsprogram.com/Data/

and contact information or URL).<br>• This text is appropriate if the data are owned by a third party and authors do not have permission to share the data.<br><br>* typeset	
Additional data availability information:	

Powered by Editorial Manager® and ProduXion Manager® from Aries Systems Corporation

Kindu Yinges Wondie

School of Midwifery

College of Medicine and Health Sciences

University of Gondar, Gondar, Ethiopia

Email: kinduyinges2010@gmail.com

Date: January 31, 2026

Editor-in-Chief

PLOS ONE

Subject: Submission of manuscript "“Content-qualified antenatal care coverage in Lesotho: An ordinal logistic regression analysis of the 2023–2024 Demographic and Health Survey”

Dear Editor,

I am pleased to submit our original research manuscript entitled “Content-qualified antenatal care coverage in Lesotho: An ordinal logistic regression analysis of the 2023–2024 Demographic and Health Survey” for consideration in *PLOS ONE*. The authors are Kindu Yinges Wondie (corresponding author), Nuhamin Tesfa Tsega, Tazeb Alemu Anteneh, Alemneh Tadesse Kassie, Berihun Agegn Mengistie, Endalk Birrie Wondifraw, and Getie Mihret Aragaw.

This study uses data from the 2023-24 Lesotho Demographic and Health Survey to examine the level and determinants of content-qualified antenatal care among 1,112 women aged 15–49 who had a live birth or stillbirth in the two years preceding the survey. We constructed ANCq as an ordinal composite score (0–16) based on timing of care, number of visits, skilled provider involvement, and receipt of essential service components. The outcome was categorized into low, moderate, and high levels. Using ordinal logistic regression, we identified significant associations with marital status, maternal education, internet use, pregnancy intention, household size, and community-level education. Our findings show that while ANC contact coverage is high in Lesotho, the quality and content of care remain uneven, underscoring the need for targeted strategies to close persistent gaps in maternal and newborn health.

This work aligns closely with *PLOS ONE*’s mission to publish rigorous and methodologically sound research that advances knowledge across disciplines. By applying a validated ANCq indicator and an ordinal modeling approach that preserves the natural ordering of care quality, our study strengthens measurement of maternal health service delivery in low-resource settings. The

use of nationally representative data enhances generalizability, and the focus on equity and service content contributes directly to efforts toward achieving Sustainable Development Goal 3.

We confirm that this manuscript is original, has not been published elsewhere, and is not under consideration by another journal. All authors have approved the manuscript and agree to be accountable for all aspects of the work. There are no conflicts of interest to declare. Ethical approval for the original survey was obtained from the ICF Institutional Review Board and the Lesotho Ministry of Health Research and Ethics Committee. We have complied with *PLOS ONE*'s policies, including data availability (the DHS data are publicly accessible) and reporting standards. We believe this manuscript will be of interest to *PLOS ONE*'s broad readership in public health, epidemiology, and health systems research. Thank you very much for considering our submission. Please direct all correspondence to me at kinduyinges2010@gmail.com.

Sincerely,

Kindu Yinges Wondie, MSc
Department of Clinical Midwifery
School of Midwifery, College of Medicine and Health Sciences
University of Gondar, Gondar, Ethiopia
Email: kinduyinges2010@gmail.com
ORCiD: 0000-0002-2769-0385

Manuscript Click here to access/download;Manuscript;manuscript.docx

Content-qualified antenatal care coverage in Lesotho: an ordinal logistic regression analysis of the 2023-2024 demographic and health survey

Kindu Yinges Wondie[1*], Nuhamin Tesfa Tsega[2], Tazeb Alemu Anteneh[1], Alemneh Tadesse Kassie[1], Berihun Agegn Mengistie[3], Endalk Birrie Wondifraw[4], Getie Mihret Aragaw[3]

[1]Department of Clinical Midwifery, School of Midwifery, College of Medicine and Health Sciences, University of Gondar, Gondar, Ethiopia; [2] Department of Women's and Family health, School of Midwifery, College of Medicine and Health Sciences, University of Gondar, Gondar, Ethiopia; [3]Department of General Midwifery, School of Midwifery, College of Medicine and Health Sciences, University of Gondar, Gondar, Ethiopia; [4]Department of Nursing, College of Medicine and Health Sciences, Debre Tabor University, Debre Tabor, Ethiopia

*Corresponding author

Email: kinduyinges2010@gmail.com (KY)

ORCiD: 0000-0002-2769-0385

Abstract

Background: Despite high antenatal care contact coverage in Lesotho, the content and quality of care remains suboptimal. This study assessed the level and determinants of content-qualified antenatal care coverage using the 2023-2024 Lesotho Demographic and Health Survey.

Methods: Data were analyzed from 1,112 women aged 15-49 who had a live birth or stillbirth in the 2 years preceding the survey. Content-qualified antenatal care was constructed as a composite score (0-16) incorporating the timing of initiation, number of contacts, attendance of skilled providers, and receipt of 10 key content components. The content-qualified antenatal

care coverage score was categorized into ordered tertiles (low: 0-12, moderate: 13, high: 14-16). Ordinal logistic regression (proportional odds model) was used to identify factors associated with higher content-qualified antenatal care categories, accounting for complex survey design.

Results: Overall, 71.9% of women had moderate or high ANCq scores. Higher content qualified antenatal care coverage was significantly associated with being married or living with a partner, having secondary or higher education (AOR = 2.05 and 1.90), using the internet at least once per week (AOR = 1.61), having a wanted pregnancy (AOR = 2.87), living in a smaller household (AOR = 1.36), and residing in a community with a higher level of education (AOR = 1.38).

Conclusion: Despite reasonable antenatal care contact coverage, content-qualified antenatal care coverage remains low and uneven. To close the coverage gap, targeted interventions to improve maternal education, internet access, pregnancy planning, and community education are essential.

Introduction

Sub-Saharan Africa (SSA) has the highest global burden of maternal deaths and stillbirths (1, 2). Lesotho has one of the highest maternal mortality ratios globally, with 478 deaths per 100,000 live births (3) and 39 infant deaths per 1000 (4). Obstetric hemorrhage, hypertensive disorders, and complications of unsafe abortion are the leading causes (5).

Antenatal care (ANC) is defined as skilled healthcare provided to women during pregnancy (6). ANC is the cornerstone of pregnancy-related complications detection, treatment, and prevention through counseling and advice, maternal and fetal assessments, vaccination, and micronutrient supplementation (7, 8).

Lesotho has made strides to end preventable maternal and newborn deaths through quality ANC (3, 5). Consequently, ANC contact coverage has improved substantially, with 93% of women attending at least one ANC contact and 82% attending at least four. However, the expected reductions in maternal and neonatal mortality were not achieved (4). This discrepancy highlights the critical quality–contact gap (9). The gap can increase because women start ANC late, do not attend the recommended number of contacts, receive incomplete care content, have scarcity of skilled providers, have inaccessible care because of geographic barriers (9-13).

Traditional ANC coverage metrics cannot measure the actual adequacy and quality of ANC coverage because they rely on the mere number of ANC contacts (14, 15). Moreover, most of them were restricted to women who had at least one ANC contact. This measure does not reflect the population-level coverage, which is essential for the coverage of maternal health services (16).

Content-qualified antenatal care (ANCq) addresses this limitation by measuring both service contact (initiation timing and number of visits) and content (receipt of core ANC components from a skilled provider) (17). In this study, ANCq is a composite score ranging from 0 to 16, where higher scores indicate coverage (16, 17). This includes first-trimester initiation, skilled provider attendance, number of contacts, and receipt of 10 key content items (blood pressure measurement, urine and blood sampling, fetal heart rate auscultation, enquiry about vaginal bleeding, counselling on diet and breastfeeding, fundal height measurement, tetanus toxoid vaccination, and iron-folic acid supplementation). ANCq has been validated as a predictor of lower mortality in neonates (18).

Despite Lesotho's health reforms, including the 2014 National Health Reform, village health worker expansion, and stronger ANC–HIV integration, the content and quality of care remains

uneven (19, 20). Traditional binary measures of “adequate” ANC mask quality gradations and exclude women with zero contacts, limiting their usefulness for tracking inequities (17).

The 2023-24 Lesotho Demographic and Health Survey (LDHS) provides data on indicators of ANC coverage at the national level (timing of initiation, number of contacts, provider skill, and essential services) (4, 16, 17). Because ANCq is an ordered outcome, ordinal logistic regression is appropriate for modeling its determinants. This approach preserves information and offers advantages over binary or multinomial models for achieving natural ordering outcomes (21). To date, no study has examined ANCq coverage or its determinants in Lesotho using the most recent 2023-2024 LDHS data, ordinal logistic regression approach been applied to capture the natural ordering of quality levels. This study addresses this gap by assessing the level of ANCq coverage and identifying factors associated with higher ANCq categories among women who had their last birth within the two years preceding the survey.

Methods and materials

Study setting

Lesotho is a small, mountainous, and landlocked country located in Southern Africa, entirely surrounded by South Africa. It covers approximately 30,000 km² and has a population of approximately 2.2 million. It is divided into ten administrative districts, namely Butha-Buthe, Leribe, Berea, Maseru, Mafeteng, Mohale’s Hoek, Quthing, Qacha’s Nek, Mokhotlong, and Thaba-Tseka, which are further subdivided into constituencies and community councils. Maseru, the capital city and the main urban center (22). The population is predominantly rural, and the mountainous terrain poses challenges to accessing healthcare services, particularly maternal and newborn care. Lesotho’s health system is decentralized and consists of hospitals, health centers, and community health posts supported by community health workers. The country has prioritized improving reproductive, maternal, newborn, child, and adolescent

health services, including the adoption of the WHO ANC recommendations for a positive pregnancy experience (3).

Data collection for the 2023-24 LDHS was conducted from November 27, 2023, to February 29, 2024 (4).

Data Sources and study design

This study is a secondary analysis of data from the 2023–2024 LDHS, a nationally representative cross-sectional survey conducted by the Ministry of Health in collaboration with international partners (4). The survey followed the DHS-8 methodology, which includes an expanded set of ANC indicators aligned with the updated ANC model of the World Health Organization (4, 23). The pregnancy and postnatal care recode file from the 2023-24 LDHS was used for this analysis (23).

The LDHS employed a two-stage, stratified cluster sampling design using the 2016 Lesotho Population and Housing Census as the sampling frame (4). Stratification was based on district and type of residence (urban, peri-urban, and rural), resulting in 29 sampling strata, as the district of Butha-Buthe does not contain a peri-urban area (4). In the first stage, 400 enumeration areas were selected as clusters using a proportional probability to size. In the second stage, all households within each selected cluster were listed, and 25 households were systematically chosen through equal probability sampling without replacement.

To account for the complex survey design, sampling weights, stratification, and clustering were applied in all analyses. The survey procedures are detailed in the 2023-24 LDHS final report (4).

Study population, sample size, and sampling procedure

The base population for this analysis was women aged 15-49 years who had their most recent live birth or stillbirth within two years preceding the survey. Data were collected through face-to-face interviews using standardized, pretested DHS questionnaires adapted to the Lesotho context and administered by trained fieldworkers (4). Of the 1,811 women aged 15-49 years, 1112 had their most recent live birth or stillbirth within the 2 years before the survey and were included in the final analysis.

Variable definition and measurement

Dependent variable

The outcome variable was ANCq coverage, a composite indicator combining service contact and content dimensions (18). The ANCq score, ranging from 0 to 16, was calculated by assigning one point for the first-trimester initiation, two points for at least one skilled provider visit, between one and three points for the number of ANC contacts (one point for 1-3 visits, two points for 4-7 visits, and three points for eight or more visits), and one point each for the receipt of ten content components, namely blood pressure measured, urine sample taken, blood sample taken, fetal heart rate listened to, asked about vaginal bleeding, counselling on maternal diet, counselling on breastfeeding, fundal height measured, at least two doses of tetanus toxoid before birth, and iron-folic acid supplementation (4, 17). The total score was categorized into three ordered levels using weighted tertiles: low (0-12), moderate (13), and high (14-16).

Independent Variables

This study assessed both the individual-and community-level variables. The variables were defined and coded according to the DHS-8 guideline (23). Maternal age was grouped into 15-24, 25-34, and 35-49 years. Marital status was coded as married or living with a partner versus not married or not in union. Educational level was categorized as no education or primary,

secondary, or higher education. Internet use in the past month was coded as none, less than once per week, and at least once per week. Pregnancy intention was coded as wanted then, wanted later, or not wanted. Household size was dichotomized as four or fewer members versus five or more members. Place of residence was coded as urban versus rural. The wealth index was categorized into quintiles (poorest, poorer, middle, richer, and richest). Community-level variables, including media exposure, literacy (proportion with secondary or higher education), and poverty (proportion in poorest or poorer quintiles), were aggregated at the cluster level and dichotomized at the median (24).

Data management and statistical analysis

Data cleaning and statistical analysis were conducted using Stata version 17. The presence and completeness of the ANCq coverage indicators and all explanatory variables were verified prior to coding and analysis. Cases with incomplete information were included in the denominator according to the DHS-8 recode guideline (23).

The dataset was weighted using sampling weights, primary sampling units, to account for the DHS complex survey design (23). The normality of the ANCq score was assessed using summary statistics, histogram inspection, and the skewness-kurtosis test. The score showed a discrete and highly skewed distribution (skewness = -2.73, kurtosis = 12.30; $p < 0.001$), with clustering at higher values and the absence of some intermediate scores (e.g., no score of 7), making treatment as a continuous outcome inappropriate. Sensitivity analysis using ANCq as a continuous score yielded similar effect directions and significance patterns, confirming the robustness of findings. Consequently, the ANCq score was categorized into three ordered levels using weighted tertiles derived from the cumulative weighted distribution.

The assumption of proportional odds for the ordinal logistic regression model was tested using the Brant (Wald) test. The test showed no evidence of violation ($\chi^2 = 0.09$, $p = 0.758$), confirming that the assumption held for all predictors and supporting the use of the model (25).

Initially, a multilevel mixed-effects ordered logistic regression was fitted to account for the clustering of individuals within enumeration areas. The null model indicated significant clustering with an intraclass correlation coefficient of 6.9% ($p < 0.001$). However, after including individual- and community-level covariates, the intraclass correlation coefficient dropped to 0.6%, and the likelihood ratio test was non-significant ($p = 0.390$). Therefore, the final model used standard ordered logistic regression with cluster-robust standard errors.

Bivariable regression analysis was performed for both individual and community-level variables. Variables with a p-value of ≤ 0.2 were considered candidates and included in the multivariable regression analysis. In the multivariable ordered logistic regression analysis, the adjusted odds ratio (AOR) with 95% confidence interval (CI) and a p-value of < 0.05 were used to declare the statistical significance and strength of determinants of ANCq coverage levels.

Ethics statement

This study was a secondary analysis of publicly available, anonymized data from the 2023-24 LDHS. Access to the dataset was granted through the DHS Program's data access portal following a formal request and approval. The protocol for the LDHS received clearance from both the ICF Institutional Review Board and the Lesotho Ministry of Health Research and Ethics Committee (4). All survey participants provided written informed consent prior to data collection; for minors, consent was obtained from a parent or legal guardian, and assent was obtained from the minors themselves (4). As this study involved secondary analysis of de-identified data, no additional ethical approval was required. The research adhered to the DHS

Program data usage policy and relevant guidelines for secondary data use (https://dhsprogram.com)

Results

Content-qualified antenatal care coverage in Lesotho

Overall, 28.2% (n = 313) of women fell into the low ANCq tertile (scores 0-12), 44.3% (n = 493) into the moderate tertile (score 13), and 27.5% (n = 306) into the high tertile (scores 14-16). Combining moderate and high categories, 71.8% of women achieved moderate or high ANCq coverage, indicating that a substantial minority (28.2%) experienced low coverage while the majority received a reasonable level of content-qualified care. The median ANCq score was 14 (interquartile range: 13-15), reflecting a skewed distribution with clustering at higher values.

Regarding service contact dimensions, 51% of women started ANC in the first trimester, 24% attended eight or more visits, while 93.2% received care from a skilled provider at least once. Individual content component coverage was generally high for blood pressure measurement and fetal heart rate assessment (95.2%) and for blood and urine sampling and fundal height measurement (94.9%). Lower coverage was observed for receipt of at least two doses of tetanus toxoid vaccination (59.2%) and counseling on maternal diet (76.5%) (Table 1).

Table 1: Content-qualified antenatal care coverage dimensions in Lesotho (n=1112)

Dimension of ANCq	Category	Frequency	Percentage
Number of ANC contacts attended	0	43	3.87
	1-3	172	15.47
	4-7	630	56.65
	≥ 8	267	24.01
ANC initiated in the first trimester	No	546	49.1
	Yes	566	50.9
Skilled provider in at least one ANC contact	No	76	6.83
	Yes	1,036	93.17
Iron-folic acid provided	No	163	14.66
	Yes	949	85.34
Blood sample	No	57	5.13
	Yes	1,055	94.87

Vaccinated with at least two doses of tetanus toxoid before delivery	No	454	40.83
	Yes	658	59.17
Urine sample taken	No	69	6.21
	Yes	1,043	93.79
Fetal heart rate assessed	No	52	4.68
	Yes	1,060	95.32
Blood pressure measured	No	53	4.77
	Yes	1,059	95.23
Fundal height measured	No	57	5.13
	Yes	1,055	94.87
Asked about vaginal bleeding	No	243	21.85
	Yes	869	78.15
Counseled about diet	No	261	23.47
	Yes	851	76.53
Counseled about breastfeeding	No	160	14.39
	Yes	952	85.61

Background characteristics of the study participants

The median age of the participants was 25 years (interquartile range: 21-31). Most women were married or living with a partner (72.7%), were not currently working (73.2%), and had attained secondary or higher education (73.3%). More than half (54.7%) reported using the internet almost every day. The majority of the respondents (61.2%) resided in rural areas, and 67.3% lived in communities with lower poverty levels (Table 2).

Table 2: Distribution of receipt of content-qualified antenatal care by background characteristics of women in Lesotho (N = 1112)

Variable	Category	Content qualified antenatal care coverage			Total (%)	P-value
		Low	Moderate	High		
Age	15-24	151	235	138	524 (43.3)	0.952
	25-34	113	189	128	430 (41.4)	
	35-49	49	69	40	158 (15.3)	
Current marital status	Married	201	335	243	779 (72.7)	0.000
	Unmarried	112	158	63	333 (27.3)	
Household head	Male	197	286	198	681 (64.5)	0.061
	Female	116	207	108	431 (35.5)	
Educational level	Up to primary	131	163	62	356 (26.8)	0.000
	Secondary	163	285	198	646 (58.7)	

	Higher	19	45	46	110 (14.6)	
Currently Working	No	252	406	224	882 (73.2)	0.009
	Yes	61	87	82	230 (26.8)	
Wealth index	Poorest	124	173	73	370 (21.7)	0.000
	Poorer	68	98	46	212 (17.2)	
	Middle	57	97	65	219 (21.8)	
	Richer	38	80	60	178 (20.5)	
	Richest	26	45	62	133 (18.8)	
Owns mobile phone	No	83	104	41	228 (16.7)	0.002
	Yes	230	389	265	884 (83.3)	
Owns bank account	No	259	374	205	838 (68.7)	0.001
	Yes	54	119	101	274 (31.3)	
Frequency of internet use	Not at all	121	160	68	349 (26.5)	0.000
	Less than once a week	30	43	13	86 (8)	
	At least once a week	32	70	40	142 (10.8)	
	Almost every day	130	220	185	535 (54.7)	
Problem to access health care	No	160	283	190	633 (64.3)	0.165
	Yes	153	210	116	479 (35.7)	
History of abortion	No	269	445	264	978 (87.6)	0.071
	Yes	44	48	42	134 (12.4)	
Number of children ever born	1	115	229	147	491 (45.1)	0.195
	2-4	168	222	145	535 (48.5)	
	≥ 5	27	39	10	76 (6.4)	
Family size	≤ 4	88	180	125	393 (38.6)	0.000
	≥ 5	225	313	181	719 (61.4)	
Last pregnancy wanted	Then	91	201	181	473 (45.7)	0.000
	Later	117	168	84	369 (31.5)	
	No more	105	124	41	270 (22.8)	
Residence	Urban	70	140	114	324 (38.8)	0.010
	Rural	243	353	192	788 (61.2)	
District of residence	Butha-Buthe	31	45	44	120 (6.6)	0.077
	Leribe	34	49	37	120 (16.7)	
	Berea	34	46	32	112 (12.1)	
	Maseru	28	55	44	127 (31.9)	
	Mafeteng	27	38	15	80 (5.6)	
	Mohale's Hoek	33	55	23	111 (6.3)	
	Quthing	27	35	21	83 (3.4)	
	Qacha's Nek	21	45	28	94 (3.4)	
	Mokhotlong	32	60	27	119 (5.3)	
	Thaba-Tseka	46	65	35	146 (8.7)	
Community-level education	Low	114	171	82	367(27.9)	0.133
	High	199	322	224	745(72.1)	
Community-level poverty	Low	146	255	197	598(67.3)	0.000
	High	167	238	109	514(32.7)	

Community-level media exposure	Low	217	328	165	710(51.9)	0.001
	High	96	165	141	402(48.1)	

Determinants of content-qualified antenatal care coverage

Several factors were significantly associated with higher ANCq categories in the multivariable ordinal logistic regression analysis (Table 3).

Women who were married or living with a partner had 60% higher odds of being in a higher ANCq category than unmarried or unpartnered women (AOR 1.60, 95% CI: 1.19-2.16). Women with secondary education (AOR 2.05, 95% CI: 1.44-2.92) and higher education (AOR 1.90, 95% CI: 1.13-3.19) had higher odds than those with no or primary education.

Women who used the internet at least once per week had 61% higher odds than those who did not use the internet (AOR 1.61, 95% CI: 1.02-2.56). Women whose last pregnancy was wanted at the time of conception had nearly three times higher odds than those whose pregnancy was not wanted (AOR 2.87, 95% CI: 2.04-4.05).

Smaller household size (≤ 4 members) was associated with a 36% higher odds (AOR 1.36, 95% CI: 1.04-1.78). At the community level, residence in clusters with a higher proportion of women who had completed secondary or higher education was associated with 38% higher odds (AOR, 1.38; 95% CI: 1.02-1.81) (Table 3).

Table 3: Bivariable and Multivariable ordinal logistic regression analysis of ANCq coverage in Lesotho

Variable	Category	COR (95% CI)	AOR (95% CI)
Current marital Status	Unmarried/not living with partner	1	1
	Married/living with partner	2.10(1.59-2.76)	1.60(1.19-2.16) **
Education level	Up to primary	1	1
	Secondary	2.19(1.64-2.91)	2.05(1.44-2.92) ***
	Higher	3.19(2.13-4.79)	1.90(1.13-3.19) *
Currently working	No	1	1
	Yes	1.59(1.20-2.12)	1.08(0.78-1.48)
Wealth Index	Poorest	1	1
	Poorer	1.06(0.72-1.55)	1.00(0.66-1.51)
	Middle	2.02(1.40-2.92)	1.56(0.94-2.60)
	Richer	2.39(1.64-3.49)	1.60(0.90-2.84)
	Richest	3.25(2.18-4.83)	1.67(0.89-3.10)
Mobile phone	No	1	1
	Yes	1.88(1.35-2.61)	0.99(0.67-1.45)
	No	1	1

Owns a bank account	Yes	1.86(1.43-2.43)	1.23(0.90-1.67)
Frequency of internet use in the past month	Never	1	1
	Less than once a week	0.71(0.44-1.14)	0.70(0.42-1.16)
	At least once a week	1.83(1.19-2.81)	1.61(1.02-2.56) *
	Almost every day	2.12(1.59-2.83)	1.31(0.91-1.87)
Problem to access health care	No	1.33(1.03-1.71)	0.94(0.71-1.24)
	Yes	1	1
Parity	1	2.00(1.21-3.30)	0.90(0.5-1.58)
	2-4	1.62(0.98-2.68)	0.66(0.38-1.14)
	≥ 5	1	1
Household size	1-4	1.76(1.37-2.27)	1.36(1.04-1.78) *
	≥ 5	1	1
Last pregnancy wanted	Then	3.86(2.81-5.31)	2.87(2.04-4.05) ***
	Later	1.47(1.06-2.03)	1.29(0.91-1.82)
	No more	1	1
Residence	Rural	1	1
	Urban	1.73(1.32-2.28)	0.95(0.66-1.35)
Community-level education	Low	1	1
	High	1.39(1.04-1.87)	1.38(1.02-1.81) ***
Community-level poverty	Low	1.89(1.44-2.47)	1.88(1.42-2.10)
	High	1	1
community-level media exposure	Low	1	1
	High	1.76(1.35-2.30)	1.37(0.98-1.91)

AOR, adjusted odds ratio; COR, Crude Odds Ratio; *, P value ≤ 0.05; **, P value ≤ 0.01; ***, P value ≤ 0.001

Discussion

This study provides the first assessment of ANCq coverage and its determinants in Lesotho using the 2023-2024 DHS. Overall, 71.9% of women achieved moderate or high ANCq scores, with a median score of 14, indicating that the full package of recommended services is not universally received although ANC attendance is high.

The magnitude of ANCq coverage in Lesotho is relatively higher than that of earlier multi-country analyses from SSA and South Asia, which reported much lower proportions using narrower content indicators or stricter binary definitions of adequacy. For example, previous studies reported low adequate ANC coverage in SSA (3%) (24), Rwanda (8.9%) (26), Tanzania (43%) (27), India (13-32%) (28-30), and Bangladesh (18%) (31). These discrepancies can be

explained by differences in measurement approaches, survey instruments, and contextual factors.

ANCq coverage was measured using an ordered scoring system that captures variation across low, medium, and high levels rather than collapsing women into only “adequate” versus “inadequate” categories. In addition, the DHS-8 questionnaire includes a broader set of ANC content indicators and applies a shorter recall period than earlier DHS phases, which may improve reporting accuracy and increase observed ANCq coverage. Differences in the number, type, and accessibility of ANC services across countries also contribute to variation in ANCq coverage. For instance, this analysis included fundal height measurement, and Lesotho’s integrated HIV-ANC programs may have strengthened the delivery of comprehensive services compared with other SSA settings.

In contrast, many previous studies (26-31) defined ANCq coverage using strict binary (adequate vs. inadequate) criteria requiring fulfilment of all recommended contacts and services for adequate ANCq coverage. This binary definition also condenses the scores to two extremes.

Several factors, including marital status, maternal education, internet use, household size, pregnancy intention, and community-level education, were significantly associated with ANCq coverage.

Marital status was significantly associated with ANCq cover, with married women or those living in union had 60% higher odds of being in a higher ANCq category compared with unmarried women. This finding is consistent with evidence from the SSASSA (32, 33). The association likely reflects the economic and social support from partners. Financial security within marriage facilitates allocation of resources to ANC, including transport, user fees, and compensation for lost income during clinic visits, while spousal involvement can enhance

women's autonomy, provide emotional support, and promote completion of recommended contacts. In Lesotho, where family and spousal structures strongly influence health-seeking behavior, these effects are amplified. Pregnancy outside marriage is often stigmatized, potentially discouraging unmarried women from engaging with ANC services (34), whereas women in unions are more likely to receive encouragement and practical assistance from partners and extended family networks (35). Evidence from culturally similar Sesotho-speaking communities shows that male involvement reduces maternal stress, improves couple communication, and promotes shared responsibility for health decisions, thereby strengthening ANC content and effectiveness (36, 37). Male partner involvement is also associated with earlier initiation, higher attendance, and better adherence to key interventions such as blood testing, contributing to improved overall ANC quality through consistent follow-up and compliance with care protocols (38).

Maternal education is a key determinant of ANCq. Women who had attained secondary or higher education had nearly twice the odds of being in a higher ANCq category than those with primary education or none. This finding is consistent with those of previous DHS analyses from Lesotho and across SSA (24, 39). The association likely reflects differences in knowledge and health literacy: more educated women are better informed about the benefits of early booking and adherence to the recommended number and content of ANC contacts, and therefore place greater value on these services. Higher educational attainment also enhances women's ability to recognize danger signs of pregnancy and understand the consequences of unqualified care (40). In the Lesotho context, education further improves women's awareness of recommended ANC components and their capacity to effectively navigate the health system, reinforcing receipt of higher ANCq (41).

Internet use has emerged as an important determinant of ANCq. Women who reported using the internet at least once per week had 60% higher odds of being in a higher ANCq category

than those who reported no use. A recent Tanzanian DHS analysis found that women who reported using internet had lower dropping out from the maternal continuum of care compared to those with no internet exposure (42). Regular internet use may expose women to maternal health information that improves their understanding of recommended services and danger signs (43). In Lesotho, where misconceptions and cultural beliefs can delay ANC care-seeking (34), access to reliable online information may help counter misinformation and support appropriate use of ANC services (35).

Pregnancy intention was also strongly associated with ANCq. Women whose last pregnancy was wanted at the time had nearly three times the odds of being in a higher ANCq category compared with pregnancies unwanted at the time of conception. This finding is consistent with DHS analyses from Rwanda, Somalia, and Bangladesh, as well as systematic reviews from Ethiopia (44-47). Unintended pregnancies are often accompanied by emotional stress, reduced motivation, and delayed engagement with health services. These factors can lead to later initiation of ANC, fewer visits, and reduced uptake of recommended service components, ultimately lowering ANC quality. Unintended pregnancies are often accompanied by emotional stress, reduced motivation, and delayed engagement with health services (47). These factors can lead to later ANC initiation, fewer visits, and reduced uptake of recommended service components, ultimately lowering ANC quality (48).

Household size showed an inverse relationship with the ANCq coverage. Women living in smaller households had higher odds of being in a higher ANCq category than those living in larger households. This finding is supported by a DHS analysis report in SSA (24). Women in larger households in extended family structures common in Lesotho may face competing domestic and caregiving responsibilities, reduced autonomy, and time constraints that limit repeated ANC attendance (49). Women with several children may also rely on prior pregnancy

experience or avoid facilities because of fear of criticism, reducing their likelihood of receiving complete ANC content (34).

Higher community-level education was significantly associated with greater ANCq coverage. Women residing in communities with higher levels of education had approximately 40% greater odds of being in a higher ANCq category compared with those from less educated communities. This finding is consistent with multilevel analyses from sub-Saharan Africa showing that community education strongly influences the quality and adequacy of antenatal care received by women (50, 51). The association likely reflects the broader social and cultural environment shaped by community-level education, which promotes maternal healthcare norms. Women are more likely to utilize antenatal care services adequately in communities with higher female literacy and more gender-equitable norms, independent of their individual socioeconomic status (52).

Conclusion

This study highlights that while most women in Lesotho attend antenatal care, many do not receive the full package of recommended services. ANCq coverage is strongly shaped by socioeconomic position and information access rather than by simple service contact alone. Marital status, maternal education, internet use frequency, pregnancy intention, household size, and community-level education were found to be significant determinants of being in a higher ANCq coverage category.

These findings indicate that, beyond contact coverage, Lesotho needs to emphasize on provision of quality and equitable ANC. Strengthening educational opportunities for women, expanding access to digital health information, and targeting disadvantaged groups are essential steps toward ensuring that all pregnant women receive comprehensive and effective antenatal care.

Strength and limitations

This study measured ANCq coverage at the population level and included a wider range of ANC contents. This helps to better understand Lesotho's progress in ensuring quality ANC. This study employed an ordinal logistic regression analysis, preserving the natural ordering inherent in the outcome variable. This prevents collapsing of ordinal data into binary categories, which would have resulted in information loss if Standard logistic regression was used. For categorical outcome variables with three or more ordered categories, such as ANCq coverage, ordered logistic regression is specifically designed and is theoretically more appropriate. Research demonstrates that "for categorical data with ordered categories, ordinal logistic regression works well" and provides advantages over other models (21).

The study used a self-reported data, which may be affected by recall or social desirability bias, although the shorter DHS-8 recall period likely minimized it. The ANC content indicators available in DHS-8 do not encompass all components recommended by the WHO 2016 ANC mode; therefore, the ANCq measure reflects only the interventions captured in the survey. Moreover, the dataset does not provide information on the timing or sequencing of individual ANC components, limiting the assessment of whether services were delivered at clinically appropriate points during pregnancy. Important facility- and system-level determinants, such as provider adherence to ANC protocols and service readiness, were not captured, restricting the examination of ANCq coverage's structural drivers.

References

1. Sexual and Reproductive Health and Research (SRH). Trends in maternal mortality 2000 to 2023: Estimates by WHO, UNICEF, UNFPA, World Bank Group and UNDESA/Population Division [Internet]. 2025 [cited 2025 Nov 12]. Available from: https://www.who.int/publications/i/item/9789240108462.
2. United Nations Inter-agency Group for Child Mortality Estimation (UN IGME).

A Neglected Tragedy: The global burden of stillbirths. United Nations Children's Fund, New York, 2020 [Internet]. [cited 2025 Dec 12]. Available from: https://www.who.int/publications/m/item/a-neglected-tragedy-the-global-burden-of-stillbirths.

3. Lesotho takes a major step towards ending preventable maternal and newborn deaths | WHO | Regional Office for Africa [Internet]. WHO | Regional Office for Africa. 2025. Available from: https://www.afro.who.int/countries/lesotho/news/lesotho-takes-major-step-towards-ending-preventable-maternal-and-newborn-deaths.

4. Ministry of Health [Lesotho] and ICF. 2024. Lesotho Demographic and Health Survey 2023–24: Final Report.

Maseru, Lesotho, and Rockville, Maryland, USA: Ministry of Health and ICF. [Internet]. [cited 2025 Nov 11]. Available from: https://www.dhsprogram.com/pubs/pdf/FR391/FR391.pdf.

5. Addressing high maternal mortality and newborn deaths in Lesotho | WHO | Regional Office for Africa [Internet]. WHO | Regional Office for Africa. 2023 [cited 2025 Nov 11]. Available from: https://www.afro.who.int/countries/lesotho/news/addressing-high-maternal-mortality-and-newborn-deaths-lesotho.

6. Guidelines Review Committee. WHO recommendations on antenatal care for a positive pregnancy experience [Internet]. 2016 [cited 2025 Nov 13]. Available from: https://www.who.int/publications/i/item/9789241549912.

7. Tunçalp Ö, Were WM, MacLennan C, Oladapo OT, Gülmezoglu AM, Bahl R, et al. Quality of care for pregnant women and newborns-the WHO vision. Bjog. 2015;122(8):1045-9.

8. Pregnant women must be able to access the right care at the right time, says WHO.

9. Carvajal-Aguirre L, Amouzou A, Mehra V, Ziqi M, Zaka N, Newby H. Gap between contact and content in maternal and newborn care: An analysis of data from 20 countries in sub-Saharan Africa. J Glob Health. 2017;7(2):020501.

10. Gill MM, Machekano R, Isavwa A, Ahimsibwe A, Oyebanji O, Akintade OL, et al. The association between HIV status and antenatal care attendance among pregnant women in rural hospitals in Lesotho. J Acquir Immune Defic Syndr. 2015;68(3):e33-8.

11. Birru E, Ndayizigiye M, Wanje G, Marole T, Smith PD, Koto M, et al. Healthcare workers' views on decentralized primary health care management in Lesotho: a qualitative study. BMC Health Services Research. 2024;24(1):801.

12. Asamani JA, Zurn P, Pitso P, Mothebe M, Moalosi N, Malieane T, et al. Health workforce supply, needs and financial feasibility in Lesotho: a labour market analysis. BMJ Glob Health. 2022;7(Suppl 1).

13. Ndayizigiye M, Allan-Blitz L-T, Dally E, Abebe S, Andom A, Tlali R, et al. Improving access to health services through health reform in Lesotho: Progress made towards achieving Universal Health Coverage. PLOS Global Public Health. 2022;2(11):e0000985.

14. Amouzou A, Leslie HH, Ram M, Fox M, Jiwani SS, Requejo J, et al. Advances in the measurement of coverage for RMNCH and nutrition: from contact to effective coverage. BMJ Global Health. 2019;4(Suppl 4):e001297.

15. Hodgins S, D'Agostino A. The quality–coverage gap in antenatal care: toward better measurement of effective coverage. Global Health: Science and Practice. 2014;2(2):173-81.

16. Amouzou A, Barros AJD, Requejo J, Faye C, Akseer N, Bendavid E, et al. The 2025 report of the Lancet Countdown to 2030 for women's, children's, and adolescents' health: tracking progress on health and nutrition. The Lancet. 2025;405(10488):1505-54.

17. Arroyave L, Saad GE, Victora CG, Barros AJD. A new content-qualified antenatal care coverage indicator: Development and validation of a score using national health surveys in low- and middle-income countries. J Glob Health. 2021;11:04008.
18. Arroyave L, Saad GE, Victora CG, Barros AJD. Inequalities in antenatal care coverage and quality: an analysis from 63 low and middle-income countries using the ANCq content-qualified coverage indicator. International Journal for Equity in Health. 2021;20(1):102.
19. Birru E, Ndayizigiye M, McBain R, Mokoena M, Koto M, Augusto O, et al. Effects of primary healthcare reform on routine health information systems (RHISs): a mixed-methods study in Lesotho. BMJ open. 2023;13(5):e071414.
20. Andom AT, Gilbert HN, Yuen CM, Mokoroane M, Ndayizigiye M, Tsoele M, et al. The impact of the Lesotho health reform in the re-structuring of the village health workers program. BMC Health Services Research. 2025;25(1):124.
21. Gambarota F, Altoè G. Ordinal regression models made easy: A tutorial on parameter interpretation, data simulation and power analysis. International Journal of Psychology. 2024;59(6):1263-92.
22. Government of Lesotho. About Lesotho - Government of Lesotho [Internet]. Government of Lesotho. 2024 [cited 2025 Nov 11]. Available from: https://www.gov.ls/about-lesotho/.
23. Croft, Trevor N., Allen, Courtney K., Zachary, Blake W., et al. 2023. Guide to DHS Statistics. Rockville, Maryland, USA: ICF.
24. Shibeshi AH, Habtie GM, Arega GG, Hussen NM, Hamed KE, Tareke SA. A multilevel analysis of receipt of adequate antenatal care and its determinants among pregnant women in 11 sub-Saharan African countries: insights from recent demographic and health surveys. Clinical Epidemiology and Global Health. 2025;36:102185.
25. Sainani KL. Multinomial and ordinal logistic regression. PM&R. 2021;13(9):1050-5.
26. Uwimana G, Elhoumed M, Gebremedhin MA, Nan L, Zeng L. Determinants of timing, adequacy and quality of antenatal care in Rwanda: a cross-sectional study using demographic and health surveys data. BMC Health Services Research. 2023;23(1):217.
27. Kessy SA, Kagoye SA, Tibenderana JR, Nyaki AY, Manongi RN, Mboya IB. Inequalities and Determinants of Quality Antenatal Care Coverage Among Women of Reproductive Age (15-49) in Tanzania: A Cross-Sectional Analysis of National Data. Health Sci Rep. 2025;8(7):e71081.
28. Ganapathy S, Soni B, Mishra A. Determinants of Quality Antenatal Care in Pregnant Women Using NFHS-4 Data: A Path Analysis Approach. Indian Journal of Community Medicine. 2025;50(3):428-36.
29. Girotra S, Malik M, Roy S, Basu S. Utilization and determinants of adequate quality antenatal care services in India: evidence from the National Family Health Survey (NFHS-5) (2019-21). BMC Pregnancy and Childbirth. 2023;23(1):800.
30. Nihal S, Shekhar C. An assessment of adequate quality antenatal care and its determinants in India. BMC Pregnancy and Childbirth. 2024;24(1):698.
31. Nurunnahar M, Hossain MP, Haque T, Rokonuzzaman SM, Pinky SD, Kairy R, et al. Quality of antenatal care and its potential impacts on delivery services and postnatal care compliance among reproductive women in Bangladesh: A situation analysis from the Bangladesh Demographic and Health Survey 2017. PLOS ONE. 2025;20(4):e0321945.
32. Tessema ZT, Minyihun A. Utilization and Determinants of Antenatal Care Visits in East African Countries: A Multicountry Analysis of Demographic and Health Surveys. Advances in Public Health. 2021;2021(1):6623009.

33. Okedo-Alex IN, Akamike IC, Ezeanosike OB, Uneke CJ. Determinants of antenatal care utilisation in sub-Saharan Africa: a systematic review. BMJ Open. 2019;9(10):e031890.
34. Letsie TM, Lenka M. Factors contributing to the late commencement of antenatal care at a rural district hospital in Lesotho. Global Journal of Health Science. 2021;13(5):32-43.
35. Lenkoane ME, Roets L, Boersema GC. Factors Motivating Early Antenatal Care Attendance in Lesotho: A Qualitative Study. Africa Journal of Nursing and Midwifery. 2025;27(1):1-19.
36. Engelbrecht M, Mulu N, Kigozi-Male G. Exploring Factors Associated with Limited Male Partner Involvement in Maternal Health: A Sesotho Socio-Cultural Perspective from the Free State, South Africa. International Journal of Environmental Research and Public Health. 2024;21(11):1482.
37. Engelbrecht M, Mulu N, Kigozi-Male G. Sesotho Women's Preferences for Male Partner Involvement During Antenatal Care and Delivery. International Journal of Environmental Research and Public Health. 2025;22(12):1867.
38. Alemi S, Nakamura K, Rahman M, Seino K. Male participation in antenatal care and its influence on their pregnant partners' reproductive health care utilization: insight from the 2015 Afghanistan Demographic and Health Survey. Journal of Biosocial Science. 2021;53(3):436-58.
39. Mkandawire P, Kangmennaang J, Walker C, Antabe R, Atuoye K, Luginaah I. Pregnancy intention and gestational age at first antenatal care visit in Lesotho. African Journal of Midwifery and Women's Health. 2021;15(1):1-11.
40. Raru TB, Ayana GM, Zakaria HF, Merga BT. Association of Higher Educational Attainment on Antenatal Care Utilization Among Pregnant Women in East Africa Using Demographic and Health Surveys (DHS) from 2010 to 2018: A Multilevel Analysis. Int J Womens Health. 2022;14:67-77.
41. Seeiso T, Todd-Maja MM. Quantifying pregnant women's knowledge of educational components of antenatal care in Lesotho. Africa Journal of Nursing and Midwifery. 2019;21(1):1-21.
42. Asnake AA, Abajobir AA, Seifu BL, Asgedom YS, Melese M, Bezie MM, et al. Multilevel analysis of dropout from maternal continuum of care and its associated factors: Evidence from 2022 Tanzania Demographic and Health Survey. PLOS ONE. 2024;19.
43. Djouma Nembot F, Buh Nkum C, Ateudjieu D, Deborah K, Ebongo Nanje Z, Ndzana R, et al. Effectiveness of a WhatsApp-based communication on improving access to antenatal care interventions in sub-Saharan Africa: A randomized control health facility trial. Digital health. 2025;11:20552076251379349.
44. Uwimana G, Elhoumed M, Gebremedhin MA, Nan L, Zeng L. Determinants of timing, adequacy and quality of antenatal care in Rwanda: a cross-sectional study using demographic and health surveys data. BMC Health Services Research. 2023;23.
45. Alam MB, Khanam S, Kabir MA, Khalif IY, Khan MN, editors. Effect of pregnancy intention at conception on the continuity of care in maternal healthcare services use in Somalia: Evidence from first national health and demographic survey. medRxiv; 2024.
46. Tolossa T, Turi E, Fetensa G, Fekadu G, Kebede F. Association between pregnancy intention and late initiation of antenatal care among pregnant women in Ethiopia: a systematic review and meta-analysis. Systematic Reviews. 2020;9.
47. Islam MH, Jubayer A, Nayan MM, Nowar A, Islam SMH. Maternal Pregnancy Intention and Antenatal Care Seeking Behaviors in Bangladesh: Evidence From Bangladesh Demographic and Health Survey, 2018. International Journal of Public Health. 2023;68.

48. Mkandawire P, Kangmennaang J, Walker C, Antabe R, Atuoye K, Luginaah I. Pregnancy intention and gestational age at first antenatal care visit in Lesotho. African Journal of Midwifery and Women's Health. 2021;15:1-11.

49. Gill MM, Hoffman HJ, Tiam A, Mohai FM, Mokone M, Isavwa A, et al. Pregnant and Postpartum Women’s Experiences and Perspectives on the Acceptability and Feasibility of Copackaged Medicine for Antenatal Care and PMTCT in Lesotho. AIDS Research and Treatment. 2015;2015(1):435868.

50. Mare KU, Sabo KG, Asgedom YS, Asmare ZA, Tebeje TM, Shibeshi AH, et al. Compliance with the 2016 WHO’s antenatal care recommendation and its determinants among women in Sub-Saharan Africa: a multilevel-analysis of population survey data. BMC Health Services Research. 2024;24(1):1223.

51. Alem AZ, Yeshaw Y, Liyew AM, Tesema GA, Alamneh TS, Worku MG, et al. Timely initiation of antenatal care and its associated factors among pregnant women in sub-Saharan Africa: A multicountry analysis of Demographic and Health Surveys. PLOS ONE. 2022;17(1):e0262411.

52. Agbaza-Mogbojuri B, Onwubuyah AO. Socioeconomic and Cultural Influences on Antenatal Care Utilization: A Multilevel Analysis. World Journal of Biology Pharmacy and Health Sciences. 2023.

Click here to access/download
Other
PLOSOne_Human_Subjects_Research_Checklist.docx
