# Supplementary material for: Content-qualified antenatal care coverage in Lesotho: An ordinal logistic regression analysis of the 2023–2024 demographic and health survey
Source: PLoS One. 2026 May 15;21(5):e0348679. doi: 10.1371/journal.pone.0348679 (PMC13178882; doi:10.1371/journal.pone.0348679)
Supplement: Attachment — Submitted filename: PLOS one review-ANCq manuscript.docx [file pone.0348679.s001.docx]

1. Line 66-67 -*ANCq has been validated as a predictor of lower mortality in neonates* (18)-A reference that buttresses the effectiveness in predicting maternal mortality is preferable since the focus is on maternal mortality.
2. Lines 68-71 do not seem to be properly placed and do not flow.
3. Line 105-114, same citation all through. Better to restructure the wording so the citation is used only once.
4. Line 73-79: Statistical analytical method is mentioned in the introduction. It should be moved to the methodology section, under data management and statistical analysis. The introduction should focus on the research gap and the study's objective.
5. Table 2: **District of residence**. Wrong calculations. Please rectify
6. Table 3: The asterisk legend is rough. Please replace with a clean legend.
7. Please can you provide a direct link to the DHS data used. I could not access the DHS 2023-2024 data as mentioned in the manuscript.
